# Gear Shape Measurement Potential of Laser Triangulation and Confocal-Chromatic Distance Sensors

**DOI:** 10.3390/s21030937

**Published:** 2021-01-30

**Authors:** Marc Pillarz, Axel von Freyberg, Dirk Stöbener, Andreas Fischer

**Affiliations:** 1 Bremen Institute for Metrology, Automation and Quality Science, University of Bremen, 28359 Bremen, Germany; a.freyberg@bimaq.de (A.v.F.); d.stoebener@bimaq.de (D.S.); andreas.fischer@bimaq.de (A.F.); 2 MAPEX Center for Materials and Processes, 330440, University of Bremen, 28334 Bremen, Germany

**Keywords:** optical gear shape measurements, lateral scanning position measurement approach, laser triangulation sensors, confocal-chromatic sensor, measurement uncertainty

## Abstract

The demand for extensive gear shape measurements with single-digit µm uncertainty is growing. Tactile standard gear tests are precise but limited in speed. Recently, faster optical gear shape measurement systems have been examined. Optical gear shape measurements are challenging due to potential deviation sources such as the tilt angles between the surface normal and the sensor axis, the varying surface curvature, and the surface properties. Currently, the full potential of optical gear shape measurement systems is not known. Therefore, laser triangulation and confocal-chromatic gear shape measurements using a lateral scanning position measurement approach are studied. As a result of tooth flank standard measurements, random effects due to surface properties are identified to primarily dominate the achievable gear shape measurement uncertainty. The standard measurement uncertainty with the studied triangulation sensor amounts to >10 µm, which does not meet the requirements. The standard measurement uncertainty with the confocal-chromatic sensor is <6.5 µm. Furthermore, measurements on a spur gear show that multiple reflections do not influence the measurement uncertainty when measuring with the lateral scanning position measurement approach. Although commercial optical sensors are not designed for optical gear shape measurements, standard uncertainties of <10 µm are achievable for example with the applied confocal-chromatic sensor, which indicates the further potential for optical gear shape measurements.

## 1. Introduction

### 1.1. Motivation

The shape of the tooth flank is of decisive importance to ensure uniform power transmission under load. In gear manufacturing, the tooth flank is often designed as an involute. However, the manufacturing of the involute tooth flank is associated with high demands on tolerances in the single-digit µm range [1]. Regardless of the gear size, deviations between the actual and the nominal involute geometry in the micrometer range already lead to defects of the gears and consequently to failures of entire gearboxes. To reduce the gearbox failure rates, the tooth shape of all teeth must be extensively measured with a measurement uncertainty in the single-digit µm range (<10 µm) [2,3,4]. Therefore, suitable position measurement systems must be provided for extensive measuring of the shape of the tooth flanks to ensure reliable gear quality inspection.

### 1.2. State of the Art

The current standard gear measurement systems are tactile coordinate measuring machines (CMM) and gear measuring instruments (GMI) [5,6]. CMM and GMI measure the tooth shape with a single-digit µm uncertainty but are limited in speed. Extensive gear shape measurements are thus time-consuming. Because of the limited speed, standard gear tests typically measure four teeth distributed around the circumference of the gear [2]. To ensure a reliable gear quality inspection, the random test scope is not sufficient. Individual tooth shape deviations from the nominal geometry, due to manufacturing tolerances or damage, are possibly not observed in standard gear tests [7]. The standard gear measurement systems are only suitable to a limited extent for extensive gear measurements.

Therefore, faster optical position sensor systems for extensive gear shape measurements of all teeth have recently been investigated [8]. In principle, optical sensors enable measurement resolutions in the nm range and offer measuring rates up to two-digit kHz. However, commercial optical sensors are typically specified for a perpendicular sensor alignment on certain flat surfaces with a defined roughness. Besides, a maximal acceptance angle is specified. To cover the entire tooth flank despite the limited accessibility through adjacent teeth, an optical sensor must be aligned nonperpendicularly to the gear surface. Accordingly, a tilt angle between the gear surface normal and the sensor axis is unavoidable, and the specified sensor uncertainty and thus the measurement uncertainty for the shape measurement in general increases with an increasing tilt angle. Furthermore, the sensors are not specified for measurements on the varying surface curvature of the tooth flanks and the surface properties of metallic gears in terms of the topography and the reflectivity (e.g., multiple reflections). It is, therefore, necessary to investigate to what extent the specifications for a perpendicular measurement arrangement on a flat surface also apply for gear shape measurements. The following described state of the art of optical approaches for gear shape measurements thus considers three fundamental challenges for optical gear metrology: the tilt angle between the surface normal and the sensor axis due to the limited optical accessibility, the varying surface curvature, and the surface properties.

Frequently investigated measurement approaches for optical gear shape measurements are based on the principle of triangulation. In 2005, Younes et al. presented an optical measurement approach for spur gear measurements with a point-by-point triangulation laser [9]. Because of the shading of adjacent teeth, the measurement does not cover the entire profile of the tooth flanks. Moreover, the achieved measurement uncertainty does not meet the requirements for gear shape measurements. Also, influences of the surface tilt, the curvature of the gear, and the surface properties are not discussed. 3D gear shape measurement principles based on laser line triangulation sensors are described by Auerswald et al. in 2019 and Guo et al. in 2020 [8,10]. Auerswald et al. investigated the laser line triangulation measurements on large helical gears. The entire tooth flank is measured and then the measurement data is compared with the reference geometry. A measurement deviation of ±8.2 µm is currently achieved. Auerswald et al. show that multiple reflections influence the measurement deviations because of the highly reflective and curved surface of the tooth depending on the sensor alignment. The potential of optical triangulation sensors for gear shape measurement is also shown by Guo et al. in 2020. For an extensive quality assessment of the tooth shape, they performed 3D measurements of the entire flank. At present, a mean measurement deviation from the ideal tooth flank of approximately −10 µm is achieved. However, it is not reported, where the remaining deviations result from. Peters et al. introduced in 2000 a fringe projector combined with a rotary table to scan the shapes of the tooth flanks of small helical gears [11]. Due to their measurement approach, they cover the entire tooth flank. The achieved measurement deviations to the theoretical tooth flank amount to approx. ±10 µm. Peters et al. explain that the sensor should be aligned as perpendicular to the tooth flank as possible to minimize measurement deviations. Influences because of the varying curvature and the highly reflective surface are not mentioned. In 2006, Meeß et al. investigated a 3D gear shape measurement approach based on stripe pattern projection [12]. They show that due to the complex gear geometry and the resulting optical accessibility, systematic length measurement deviations occur in fringe projection. Deviations to the reference geometry of approx. ±10 µm are determined after calibration. Area-oriented gear shape measurement approaches using projection moiré are presented in [3,13]. In 2005, Sciammarella et al. measured the profile contour of an entire tooth and achieved a measurement uncertainty <3.5 µm after a calibration of the measurement set-up. Challenges due to the tilt angle between the surface normal and the sensor axis, the varying curvature, and the surface properties are not described. Chen et al. presented in 2019 3D measurements of moiré projection on a small spur gear. Here, too, the entire tooth flanks are measured. The measurement deviations amount to <5 µm and uncertainty contributions due to the surface tilt, the curved geometry, and the surface properties are also not discussed. In summary, the current triangulation approaches for optical gear shape measurements indicate a great potential for a fast and extensive gear shape quality inspection. However, a detailed characterization of the remaining measurement deviations from the reference geometry concerning the aforementioned challenges (the surface tilt, the curved involute geometry, and the surface properties) is still pending.

Further investigated optical gear shape measurement approaches are based on interferometry. Fang et al. measured the geometry of a tooth flank in 2014 and after compensation of installation deviations, the measurements are in good agreement with the reference geometry [14]. However, the deviations are not further specified and not discussed in terms of the challenges in optical gear shape metrology. Balzer et al. used in 2015 and 2017 a frequency-modulated interferometric sensor [2,7]. They combined the interferometric sensor with a CMM by integrating the beam path via fiber-optics into the sensor head of the CMM. Information on deviations between measured and reference flank is not given.

Confocal-chromatic distance sensors are another promising optical sensor concept for measuring gear shapes because they are typically specified as surface independent and distance uncertainties in the low single-digit micrometer range are claimed to be achievable [15]. Consequently, the reflecting and curved tooth flanks are measurable. First measurements on small spur gears showed that measurement deviations from the reference geometry of ±10 µm are achievable [16]. The reason for the deviations was primarily the motion control unit of a rotary table on which the gear to be measured was positioned to enable the continuous detection of the tooth flanks. Influences due to the varying curvature and surface properties were mentioned but not characterized in detail. Moreover, because of a perpendicular alignment of the sensor to the tooth flank, not the entire tooth flank could be detected. Hence, further investigations are necessary to understand the potential of confocal-chromatic sensors for optical gear shape measurements.

In summary, measurement uncertainties of <10 µm from the reference geometry are achievable when optically measuring the tooth shape of a gear. However, the contributions to the measurement deviations resulting from the tilt angle between the gear surface normal and the sensor axis, the varying curvature as well as the metallic surface properties like the topography and the reflectivity (multiple reflections) were not yet discussed in detail. Therefore, the full potential of optical sensors for gear shape measurements is not known.

### 1.3. Aim and Structure of the Article

To understand the potential of triangulation and confocal-chromatic sensors in particular for the application on gears, this article presents gear shape measurements using an optical position measurement approach with a commercial confocal-chromatic and a commercial laser triangulation sensor, respectively. In particular, three potential sources of deviations are considered: the tilt angle between the gear surface normal and the sensor axis, the varying curvature as well as the metallic gear surface properties (primarily the topography). Based on the position measurements, the achievable standard measurement uncertainty for the tooth shape is to be determined. For this purpose, measurements on an involute tooth flank standard are performed. Besides, measurements on a small involute spur gear are examined to also qualitatively characterize the effect of multiple reflections due to the metallic reflective gear surface on the measurement result.

Section 2 introduces the principle of the optical position measurement for the tooth shape measurement together with the model-based evaluation for calculating the standard measurement uncertainty. In Section 3 the used components and the realized experimental set-up for the optical gear shape measurement using a commercial laser triangulation sensor and a commercial confocal-chromatic sensor, respectively, are described. Note, the operating modes of the sensors are not described in detail, as they are considered to be common knowledge. Section 4 presents and discusses the experimental results. After investigating the influence of each potential source of deviation on the measurement results, the finally achievable standard measurement uncertainty due to random sources is determined for both sensors. Section 5 closes the article with a conclusion and outlook.

## 2. Measurement Principle

### 2.1. Gear Shape Measurement Principle Using Optical Position Measurements

Two optical position measurement approaches are investigated concerning their applicability for measuring the shape of tooth flanks. Together with the description of each approach, the respective sensor requirements regarding the measuring range and the acceptance angle are compared. Note that in addition to the sensor specifications, the optical accessibility, which depends on the gear geometry, may also limit the measurement. Note further that the article focuses on nonmodified involute spur gears. The involute describes a curve traced by a point when unwinding a thread on a base circle. As the thread is unwound, the curvature of the resulting involute decreases steadily. The shape of the involute depends on the radius of the base circle. With an increasing base circle radius, the general curvature decreases.

**Figure 1:**
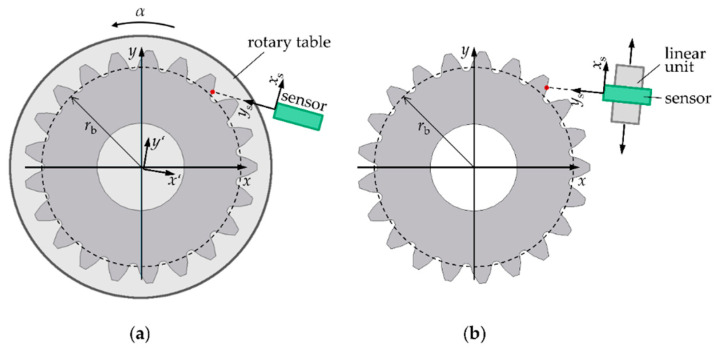
Optical position measuring principles for gear shape measurements consisting of (**a**) an optical distance sensor (*x*_s_, *y*_s_) in combination with a rotary table (*x’*, *y’*) which continuously measures the tooth contour of a gear (*x*, *y*) as a function of the rotation angle *α* and (**b**) an optical sensor mounted on a linear unit to a measuring unit (*x*_s_, *y*_s_) for laterally scanning the tooth contour of a gear (*x*, *y*).

Figure 1a shows the position measurement approach consisting of an optical distance sensor in combination with a rotary table for rotational scanning. The gear is concentrically mounted on the rotary table. The sensor alignment can be variably adjusted depending on the desired tooth flank region to be measured. Considering an involute tooth shape, a measurement perpendicular to the gear surface is possible if the sensor is aligned tangentially to the base circle (dashed line) of the gear. Therefore, optical sensors with a small acceptance angle are in principle usable for gear measurements. However, to optically access the entire tooth flank, the sensor must be turned in the direction of the gear center. A continuously changing tilt angle between the gear surface normal and the sensor axis then occurs during the scanning with the gear rotation, and the maximum tilt angle occurs at the tooth root. As the gear rotates, the shape of each tooth flank is continuously measured in form of distances *d*_i_ in the *y*_s_-direction of the sensor coordinate system (*x*_s_, *y*_s_) over the rotation angles *α*_i_, where *i* denotes the number of the distance measurement.

This measurement approach requires an optical sensor with a comparably large measuring range. For a gear with a normal module of 10.64 mm, 20 teeth, and a base circle radius of approx. 100 mm, for example, an optical distance sensor with a maximum acceptance angle of approx. ±30° and a measuring range of >25 mm is required to measure the entire tooth flank.

Another position measurement approach for gear shape measurements is shown in Figure 1b. It consists of an optical sensor that is mounted on a linear unit for scanning. The optical sensor is positioned perpendicular to the scanning direction of the linear unit. Both the optical sensor and the linear unit form a scanning measuring unit. The alignment of the measuring unit can be variably adjusted according to the tooth flank region to be measured and the maximum acceptance angle of the distance sensor. Here too, the optical sensor must be turned in the direction of the gear center to measure the entire tooth flank. The tooth flank is scanned by the lateral movement of the measuring unit and the measurement data are also stored in the form of coordinates *P*_s,i_ = (*x*_s,i_, *d*_i_) in the measuring coordinate system. Note that according to Figure 1b, only the shape of one tooth is measured. To measure the tooth shape geometry of all teeth, a rotary table is necessary for the sequential positioning of each gear tooth in the measurement region. In order to characterize the potential of an optical sensor for the application on gears, however, the measurement of one tooth flank is sufficient. Due to the laterally scanning measuring strategy and the involute tooth geometry, a continuously changing tilt angle between the gear surface normal and the sensor axis occurs. If the entire tooth flank is to be measured, the maximum tilt angle occurs at the tooth root.

In comparison with the rotational scanning approach, the laterally scanning approach requires a similar acceptance angle but a significantly smaller measuring range to measure the entire tooth flank. For the shape measurement of a gear with a normal module of 10.64 mm, 20 teeth, and a base circle radius of approx. 100 mm, the required acceptance angle is still approximately ±30° while the required measuring range amounts to only 4 mm. For this reason, only the position measurement approach with lateral scanning is considered here further.

### 2.2. Assessment of the Gear Shape Standard Measurement Uncertainty

In order to determine the measurement uncertainty of the optical gear shape measurement, the measured gear geometry needs to be compared with reference data. For this purpose, the measured distances and the reference gear geometry must be transferred to a common coordinate system. Here, the workpiece coordinate system (*x*, *y*) is selected. To transfer the sensor data to the workpiece coordinate system by mathematical transformations, the position and the alignment of the sensor relative to the workpiece coordinate system, respectively the exact transformation between the sensor coordinate system (*x*_s_, *y*_s_) and the workpiece coordinate system should be known, which is not the case here. Assuming that the center of the gear is ideally located in the center of the workpiece coordinate system, the measurement data is fitted to the reference geometry. For this purpose, a geometric model of the gear is required.

**Figure 2:**
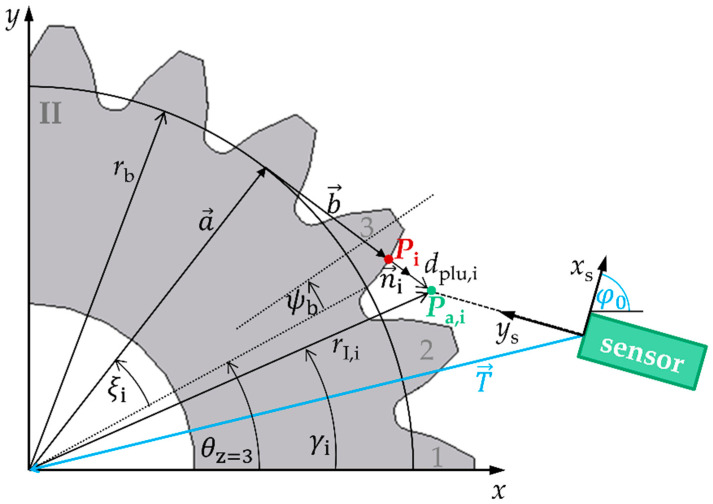
Geometric model of a nonmodified involute gear for calculating the plumb line distance *d*_plu,i_ between the measured and the nominal geometry based on a measured actual point *P*_a,i_ on tooth *Z*, the base point *P*_i_ of the nominal geometry for the plumb line distance and the position parameters $\xi_{i},\theta_{z},\psi_{b},\vec{T},\varphi_{0},r_{I,i},\gamma_{i},{\vec{n}}_{i}$ in the workpiece coordinate system. The plumb line distance between the measuring point and the nominal geometry of the tooth flank is displayed enlarged. Figure 2 is modified according to [20].

Nonmodified involute spur gears are considered here, and their nominal gear geometry is used as a reference. In Figure 2, this nominal geometry in the transverse section is shown in a workpiece coordinate system (*x*, *y*) that is shifted and rotated to the sensor coordinate system (*x*_s_, *y*_s_). The nominal geometry of involute spur gears can be described by geometry and position parameters employing the geometric model according to [17,18,19]: A nominal point *P*_i_

(1) $$P_{i}=\left[\begin{array}{c} x_{p,i}\\ y_{p,i} \end{array}\right]=\vec{a}+\vec{b}=r_{b}\cdot \left[\begin{array}{c} \cos\left(\xi_{i}+\theta_{z}-\psi_{b}\right)\\ \sin\left(\xi_{i}+\theta_{z}-\psi_{b}\right) \end{array}\right]+r_{b}\cdot \xi_{i}\cdot {\left[\begin{array}{c} \sin\left(\xi_{i}+\theta_{z}-\psi_{b}\right)\\ -\cos\left(\xi_{i}+\theta_{z}-\psi_{b}\right) \end{array}\right]}_{\;}$$

on the tooth flank of tooth Z results from an addition of a radial vector $\vec{a}$ and a tangential vector $\vec{b}$ in the workpiece coordinate system (*x*, *y*). The vector $\vec{a}$ has the length of the geometry parameter base circle radius *r*_b_ of the spur gear while the angle $\delta_{i}=\xi_{i}+\theta_{z}-\psi_{b}$ depends on position parameters $\xi_{i},\theta_{z},\psi_{b}$. The length of the tangential vector $\vec{b}$ amounts to $r_{b}\cdot \xi_{i}$ with a corresponding angle of $\delta_{i}-\frac{\pi }{2}$. The parameter $\xi_{i}$ describes the rolling angle of the spur gear assigned to the nominal point *P*_i_. The center axis of a tooth Z is defined by the angle *θ*_z_ and the base tooth-thickness half-angle by the angle $\psi_{b}$.

Because a measured point *P*_s,i_ = (*x*_s,i_, *d*_i_) is acquired in the sensor coordinate system (*x*_s_, *y*_s_), a translation vector $\vec{T}={\left[x_{t},y_{t}\right]}^{T}$ as well as a rotation angle $\varphi_{0}$ to the workpiece coordinate system must be added

(2) $$P_{a,i}=\left[\begin{array}{c} x_{a,i}\\ y_{a,i} \end{array}\right]=\;R\left(\varphi_{0}\right)\cdot (P_{s,i}+\vec{T})$$

If deviations between the optically measured actual points *P*_a,i_ and the nominal points *P*_i_ in workpiece coordinate system occur, they can be described by the plumb line distance *d*_plu,i_. A measured point *P*_a,i_

(3) $$P_{a,i}=\;P_{i}+\frac{d_{\mathrm{plu},i}}{\left|{\vec{n}}_{i}\right|}\cdot {\vec{n}}_{i}$$

is therefore composed of the addition of the nominal point *P*_i_ and the plumb line distance, which is oriented in the normal direction ${\vec{n}}_{i}$

(4) $${\vec{n}}_{i}=\left[\begin{array}{c} \sin\left(\xi_{i}+\theta_{z}-\psi_{b}\right)\\ -\cos\left(\xi_{i}+\theta_{z}-\psi_{b}\right) \end{array}\right]$$

to the surface of the nominal geometry. To characterize the gear shape measurements using the position measurement approaches realized with a laser triangulation and a confocal-chromatic sensor, respectively, the plumb line distances must be determined. Therefore, the inverse problem

(5) $$d_{\mathrm{plu},i}=f\left(P_{a,i}\left(x_{s,i},d_{i},\vec{T},\varphi_{0}\right),r_{b},\xi_{i},\theta_{z},\psi_{b}\right)$$

must be solved. Note that the transformation parameters between the sensor and workpiece coordinate system ($\vec{T},\varphi_{0}$) as well as the position parameter $\xi_{i}$ are unknown. When solving the inverse problem, the unknown parameters are also calculated. The parameters ($r_{b},\theta_{z},\psi_{b}$) result from the nominal geometry of the gear as well as from the measurement conditions.

In order to solve the inverse problem, the geometric model of the nominal involute is fitted to the measured data by iteratively minimizing the sum of the squared plumb line distances

(6) $$\begin{array}{c} \min\\ \xi_{i},\;\;x_{t},\;\;y_{t},\;\varphi_{0} \end{array}\left(\overset{k}{\underset{i=1}{\sum }}d_{\mathrm{plu},i}^{2}\right),$$

i.e., by using a nonlinear least-squares method. According to [17,21], the plumb line distances to the nominal involute of a spur gear are determined by

(7) $$d_{\mathrm{plu},i}=r_{b}\cdot \left(\sqrt{\frac{r_{I,i}^{2}}{r_{b}^{2}}-1}-fladir\cdot \left(\gamma_{i}-\theta_{z}+\psi_{b}-\varphi_{0}+\arctan\left(fladir\cdot \sqrt{\frac{r_{I,i}^{2}}{r_{b}^{2}}-1}\right)\right)\right).$$

The appropriate rolling angles $\xi_{i}$

(8) $$\xi_{i}=\sqrt{\frac{r_{I,i}^{2}}{r_{b}^{2}}-1}$$

of the root points of the plumb line distances on the involute are determined implicitly by $r_{I,i}$ and $r_{b}$. The parameter $r_{I,i}$

(9) $$r_{I,i}=\sqrt{{\left(x_{a,i}-x_{t}\right)}^{2}+{\left(y_{a,i}-y_{t}\right)}^{2}}$$

describes the radius of the measured actual point *P*_a_ in polar coordinates in the workpiece coordinate system. The parameter $\gamma_{i}$

(10) $$\gamma_{i}=\arctan\left(\frac{y_{a,i}-y_{t}}{x_{a,i}-x_{t}}\right)$$

is the corresponding polar angle to $r_{I,i}$. The symbol “*fladir*” defines a factor for the flank side (left side: −1, right side: 1).

**Figure 3:**
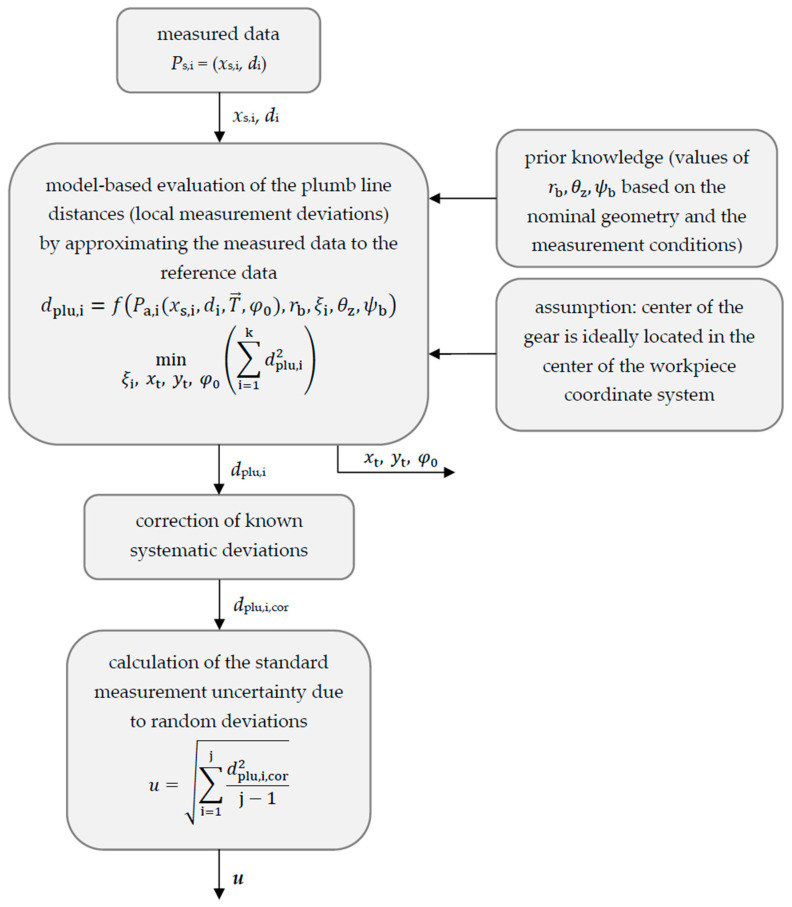
Procedure for determining and assessing the gear shape standard measurement uncertainty.

The standard measurement uncertainty for the gear shape measurement can be assessed based on the plumb line distances. The assessment procedure is shown in Figure 3. The plumb line distances represent the local measurement deviation between the measured geometry and the reference geometry. The calculated local measurement deviations are influenced by systematic and random effects. Because systematic deviations can be corrected by calibration measurements, the potential of optical sensors for gear shape measurements is mainly determined by the occurring random deviations. Therefore, in this article, the standard deviation of the random deviations is evaluated as a measure of the gear shape standard measurement uncertainty.

## 3. Experimental Set-Up

To characterize the potential of triangulation and confocal-chromatic sensors for optical gear shape measurements, the lateral scanning position measurement approach from Figure 1b is realized. Note that the particular aim of the experiments is to characterize the influence of the tilt angle between the gear surface normal and the sensor axis, the varying curvature as well as the gear surface properties like the topography and the reflectivity (multiple reflections) on the gear shape measurement results. For this purpose, measurements on a tooth flank standard as well as on a small spur gear with nonmodified involute tooth flanks are performed.

### 3.1. Measurement Objects

**Figure 4:**
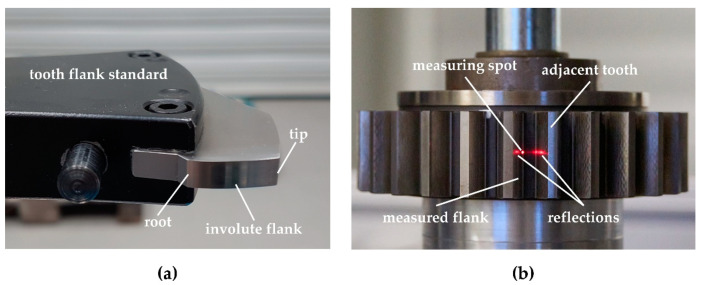
Measuring objects for evaluating the potential of the triangulation sensor and confocal-chromatic sensor for gear shape measurements and for investigating the measurement deviation contributions through the tilt angle between the gear surface normal and the sensor axis, the varying surface curvature, and gear surface properties. (**a**) shows an involute tooth flank standard with a nominal geometry with a normal module of 10.64117 mm, 20 teeth, and a base circle diameter of 199.99 mm. (**b**) shows a spur gear with involute profile and a nominal geometry with a normal module of 3.75 mm, 26 teeth and a base circle radius of 91.62 mm. In addition, the measuring spot and multiple reflections of a triangulation measurement are visible on the spur gear.

The two measurement objects are an involute tooth flank standard and a small spur gear, see Figure 4. Both measurement objects have a metallic ground surface. The parameters that describe the nominal geometries are listed in Table 1. Because the geometry of each measurement object is well-known, no measurement artifacts due to surface defects are expected to occur.

The tooth flank standard is a free-standing tooth flank and is therefore easily accessible for the optical sensors, see Figure 4a. No adjacent teeth restrict the measuring arrangement so that the entire involute of the tooth flank is measurable with a minimal sensor acceptance angle in addition to a small measuring range. With an optimal arrangement of the optical sensor, tilt angles <18° are possible both at the tooth root and at the tooth tip. The curvature decreases monotonically from the root ($0.16\frac{1}{\mathrm{mm}}$) to the tip ($0.02\frac{1}{\mathrm{mm}}$) of the tooth. The tooth flank standard is therefore particularly suitable for investigating the effect of the tilt angle between the gear surface normal and the sensor axis as well as the effect of the varying tooth flank curvature on the measurement deviation. Furthermore, the measurement deviation contribution due to the surface properties can be examined concerning the topography and the surface scattering/reflection behavior, separately from disturbances due to multiple reflections. Because the tooth flank normal is free-standing, no multiple reflections from adjacent teeth occur.

A small spur gear is used in addition to the tooth flank standard to examine the effect of multiple reflections due to the metallic, highly reflective surface and the adjacent teeth on the measurement results. The spur gear is shown in Figure 4b. To quantify the effect of multiple reflections, comparison measurements are performed on one tooth with and without covering the adjacent tooth flank with a layer of black carbon that reduces the light reflections. Here, adjacent teeth restrict the sensor alignment to the tooth flank.

### 3.2. Measurement Arrangement

**Figure 5:**
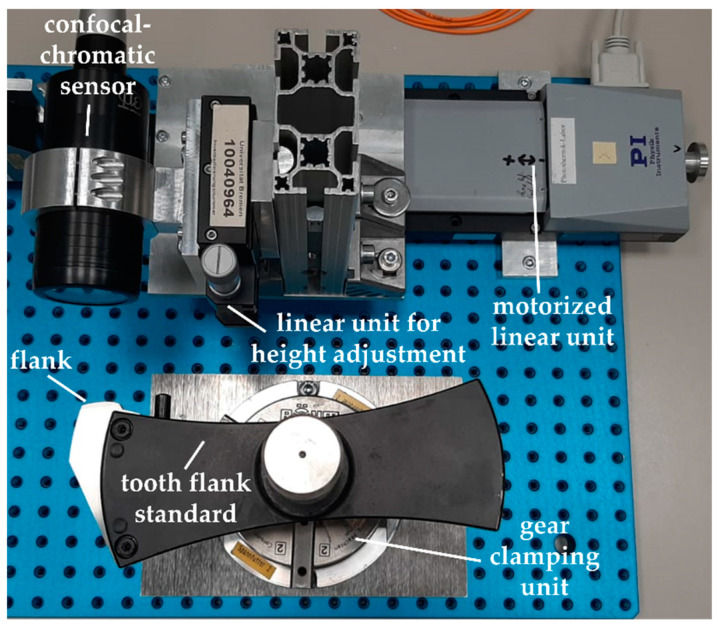
Experimental set-up of the optical position measurement approach for the gear shape measurement with a linear unit for laterally scanning the tooth flank of a tooth flank standard and using the confocal-chromatic sensor as an example. An additional linear unit allows the height of the optical sensor to be adjusted. To examine the influence of multiple reflections, the tooth flank standard is exchanged with a spur gear.

Figure 5 shows the experimental set-up of the realized lateral scanning position measurement approach according to Figure 1b. As an example, the set-up is shown for the gear shape measurements on the tooth flank standard using the confocal-chromatic sensor. As an optical distance sensor, a confocal-chromatic sensor and a laser triangulation sensor are used, respectively.

The optical distance sensor is mounted as perpendicular as possible to the scanning direction of the motorized linear unit. The linear unit is specified with a position uncertainty of 1 µm per 25 mm traveling distance. Both the sensor and the linear unit form the scanning measuring unit. An additional linear unit enables the height adjustment of the optical sensor.

The tooth flank standard and the small spur gear are mounted in front of the optical sensor with an appropriate gear clamping unit, respectively. The measuring unit and the tooth flank to be measured are aligned with each other with the aim of measuring the maximum gear tooth region. Regarding the free-standing tooth flank standard, the measuring unit is slightly turned in the direction of the gear center until the size of the tilt angle between the surface normal and the sensor axis is almost the same at both the tooth tip and the tooth root. If the tilt angle at the tooth tip exceeds the acceptance angle of the sensor, the sensor is turned back step by step in order to measure the shape of the tip. When measuring the spur gear, the measuring unit is oriented past the adjacent tooth in the direction of the tooth root until the maximum acceptance angle is accomplished. The measurement objects are then positioned in the middle of the measuring range of the optical sensor. Further, the sensor axis must be arranged in the transverse section of the measured gear. Deviations from a perpendicular sensor adjustment to the scanning movement and a sensor axis alignment in the transverse section of the gear lead to a geometrically distorted measurement result. The measuring unit then laterally scans the shape of the tooth flank.

As a result of the chosen measurement arrangement, the entire tooth flanks of the tooth flank standard and the spur gear are measurable within a measuring range of 4 mm, and only the acceptance angles of the optical sensors limit the measurable gear tooth region. On the tooth flank standard, the maximum occurring tilt angle between the surface normal and the sensor axis amounts to approx. 18°. When measuring the entire flank of the small spur gear, a maximum tilt angle of 36.7° occurs.

### 3.3. Specifications of the Optical Distance Sensors

The specifications of the applied laser triangulation sensor and the confocal-chromatic sensor are listed in Table 2. Note that the reproducibility of the confocal-chromatic distance sensor is not specified in the datasheet and thus determined experimentally. The listed sensor parameters are subsequently discussed concerning the deviation sources in the order: (1) tilt angle between the gear surface normal and the sensor axis, (2) varying curvature, (3) gear surface properties.

The laser triangulation distance sensor is specified for a white, diffuse reflecting ceramic surface. The measuring range is 50 mm and the acceptance angle amounts to ±30°. Hence, considering the geometry of the free-standing tooth flank standard, the entire flank is measurable. The measurement of the entire tooth flank of the spur gear is not feasible due to the required acceptance angle of ≈36.7°. Approximately 83% of the gear tooth range from tip to root is measurable according to the sensor specifications. Moreover, the datasheet specifies a possible measurement deviation due to the measurement of tilted surfaces. The exact value of the measurement deviation depends on the surface properties of the measurement object. The light spot size and thus the lateral resolution varies over the measuring range between 55 µm to 570 µm. In the middle of the measuring range, the light spot has the smallest diameter. Due to the surface tilt and the varying curvature of the tooth flank, a distance variation occurs within the measuring spot. Depending on the factory-made signal processing of the triangulation sensor, the distance variation within the spot can lead to the tilt angle-dependency and further a curvature-dependent measurement deviation contribution. As a result, a deterministic systematic effect is assumed, respectively, because the tilt angle, as well as the curvature, varies continuously when measuring the tooth flank.

The laser triangulation sensor is characterized by a linearity error of ±30 µm, but an exact characteristic curve of the linearity error depending on the measured distance is not known. It is assumed that a part of the linearity error on the measurement deviations of the gear shape measurements depends on the random local surface properties. Therefore, a random deviation contribution to the achievable measurement uncertainty is expected.

The specified reproducibility of the laser triangulation sensor is ±2 µm, which represents the random measurement deviation of the sensor. Measurement deviations for repeated measurements are therefore not significant compared to the expected systematic deviations due to surface tilt, the surface curvature, and the surface properties.

The confocal-chromatic distance sensor is characterized by a flat and reflective glass surface. The measuring range is 10 mm and the maximum permissible acceptance angle is ±17°. According to the geometry of the tooth flank standard, the specified acceptance angle of the confocal-chromatic sensor limits the measurable gear tooth region. Theoretically, 97.5% of the gear tooth region from tip to root is measurable. Also, the measurable tooth flank region of the spur gear is restricted due to the specified acceptance angle. The maximum measurable tooth flank region amounts to 60% (from tip to root). The datasheet describes that a possible measurement deviation emerges when the surface is tilted to the sensor axis, but this deviation is not further quantified. The light spot size of the confocal-chromatic sensor and thus the lateral resolution amount to 16 µm. Similar to the triangulation sensor, the surface tilt and the curvature of the tooth flank also lead to a distance variation within the measuring spot. In dependence on the signal processing of the confocal-chromatic sensor, a deterministic effect on the measurement deviation is therefore possible.

The linearity error of the confocal-chromatic sensor amounts to ±2.5 µm in the measuring range. Here too, an exact characteristic curve of the linearity error behavior of the sensor is not known. It is expected that this influence on the measurement deviation is also dependent on the random local surface properties of the gear. Typically, confocal-chromatic sensors are known as surface-independent. However, a random deviation contribution to the measurement uncertainty is awaited. The extent to which the tooth shape measurement is influenced by the surface properties is not apparent from the sensor specifications.

A reproducibility value is not specified for the confocal-chromatic sensor. Thus, the value for the reproducibility is determined experimentally to ±0.3 µm. Here too, the measurement deviations for repeated measurements are significantly smaller in comparison to the assumed systematic deviations due to the surface tilt, the surface curvature, and the surface properties.

### 3.4. Experimental Test Series

The influence of the tilt angle between gear surface normal and the sensor axis, as well as the varying curvature of the tooth flank on the measurement deviation are examined by comparing the measured gear shape with the reference geometry of the tooth flank standard. Based on the fitted measurement data the tilt angles and the local curvature are estimated. Note that the influence of the tilt angle and the surface curvature are superimposed during the gear shape measurement. The absolute value of the tilt angle varies almost symmetrically around the center of the measured flank whereas the surface curvature decreases monotonically from the root to the tip of the flank. Hence, it is assumed that two different plumb line deviations have to be observed for each absolute tilt angle if the surface curvature has a significant influence on the measurement deviations.

**Figure 6:**
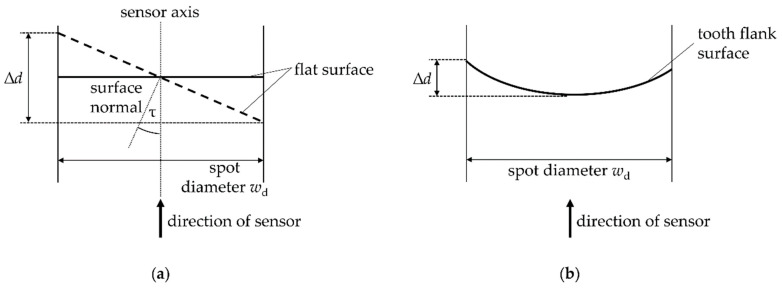
Principle sketch for the theoretical estimation of the distance variation ∆*d* within the measuring spot with a diameter of *w*_d_ of the optical distance sensors resulting from the tilt angle *τ* between tooth surface normal and sensor axis and the curvature of the tooth flank. (**a**) shows the influence of the tilt angle simplified on a flat surface. (**b**) shows the influence of the curvature of the tooth flank for a gear geometry corresponding to the tooth flank standard.

In order to estimate the magnitude of both effects, a theoretical contemplation of the geometrical influence of the tilt angle and the surface curvature depending on the lateral resolution of the sensors is carried out. When measuring a tilted surface (see Figure 6a) or a curved surface (see Figure 6b) a distance variation ∆*d* occurs within the light spot of the sensor. For the estimation of the geometrical influence of the tilt angle according to Figure 6a, a tilt angle of *τ* = −18° is applied. The results of the theoretical contemplation are shown in Table 3. In the middle of the measuring range of the triangulation sensor, a distance variation of 17.9 µm occurs within the measuring point. For the confocal-chromatic distance sensor, the distance variation within the measuring spot amount to 5.2 µm.

The occurring distance variations within the respective measuring spots due to the curvature are three orders of magnitude smaller than the estimated distance variations resulting from the surface tilt. Regarding these theoretical results, no significant influence of the surface curvature on the plumb line deviations is expected. Only the tilt angle can lead to observable deviations, which should be independent of the angle sign.

The assumed random deviation contribution due to the surface properties, especially of the peculiarity of the random local topography on the measurement deviations, is investigated by comparison measurements of the shape of the tooth flank standard at different heights. Except for the measuring position in the height, the measuring conditions are kept constant. Thus, the occurring tilt angles between the surface normal and the sensor axis as well as the occurring local curvatures are nearly the same during the comparison measurements. Merely the local surface topography changes. Hence, with the comparison measurements, the interaction of the optical sensor principles with the surface topography and the potential effect on the measurement deviation can be studied.

The gear shape measurement of the free-standing tooth flank standard lacks the consideration of the possible effect of surface-dependent multiple reflections from adjacent teeth on the measurement uncertainty. Therefore, this effect is investigated with comparative shape measurements on a small spur gear with and without covering the adjacent tooth flank with a black carbon layer. The black carbon layer is used to reduce the specular light reflections because black carbon absorbs usually more than 95% of the incident light. During the comparison measurements, the measurement conditions are not changed except for the additional black carbon layer. Thus, the same points of the gear surface are detected and the effect of the multiple reflections on the measurement deviations can be determined.

## 4. Results

### 4.1. Tooth Flank Standard Measurements

In the following, the measurement results on the tooth flank standard are presented and studied concerning the potential systematic deviation sources: surface tilt, varying curvature, and surface properties. In the end, in order to quantify the potential of the sensors for optical gear shape measurements, the standard measurement uncertainty due to random deviations is estimated. Note that the total uncertainty is also influenced by the systematic deviation sources like, e.g., the influence of the surface gradient or the alignment of the sensor and the linear unit. Therefore, the total uncertainty can increase significantly, if these systematic influences are not thoroughly eliminated by precise alignments and calibration measurements.

#### 4.1.1. Systematic Deviations

**Figure 7:**
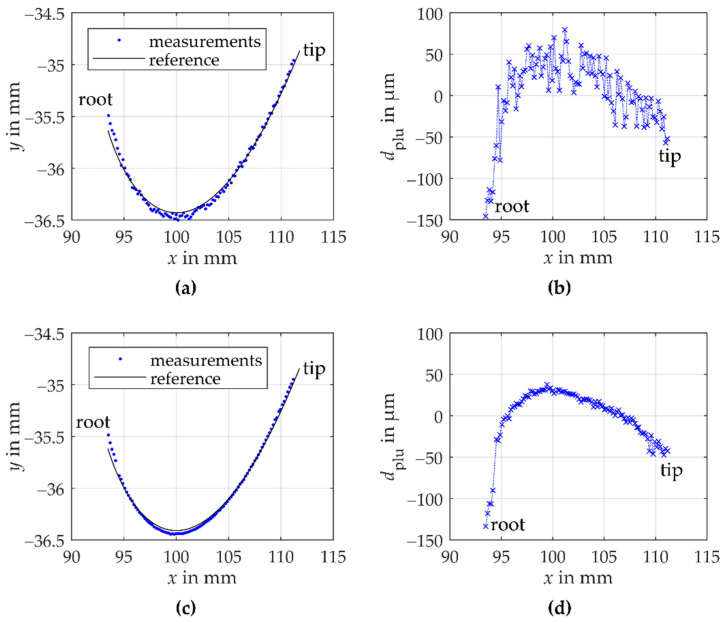
Results of one gear shape measurement with the triangulation sensor and confocal-chromatic sensor on the involute tooth flank standard, respectively. (**a**) shows the transformed triangulation measurement points as blue dots in comparison to the reference geometry of the tooth flank as a black line in a common coordinate system. Note that the x- and *y*-axis are scaled differently. (**b**) shows the plumb line distances between the reference and the measured geometry of the tooth flank plotted over the x-positions of the laser triangulation measurement points. (**c**) illustrates the transformed confocal-chromatic measurement points as blue dots compared to the reference geometry of the tooth flank as a black line in a common coordinate system. (**d**) illustrates the plumb line distances between the reference and measured geometry of the tooth flank depending on the x-positions of the confocal-chromatic measurement points.

The tooth flank is scanned laterally step by step with a lateral resolution of 0.175 mm. Per measuring position 100 distance measurements are performed and a total of 102 measuring positions is recorded with both the laser triangulation and the confocal-chromatic sensor. Figure 7 shows the results of one laser triangulation gear shape measurement (see Figure 7a,b) and one confocal-chromatic gear shape measurement (see Figure 7c,d).

In Figure 7a, the fitted laser triangulation measurement data to the reference geometry of the tooth flank standard in the workpiece coordinate system are shown. The blue dots illustrate the measurements and the black line the reference involute. Figure 7b illustrates the corresponding plumb line distances between the reference geometry and the measured geometry depending on the *x*-component of the measurement points according to Equation (6). With the laterally scanning position measurement approach using the triangulation sensor, the entire tooth flank is measurable. The curvature of the measurement data deviates from the reference geometry of the involute tooth flank standard. In the area of the root as well as in the area of the tip, the measured involute is more curved than the reference geometry. Figure 7b illustrates the deviation of the measurement data compared to the reference geometry of the tooth flank. The plumb line distances systematically deviate with a parabolic trend from the reference tooth flank in an order of magnitude of 100 µm. Moreover, the measurement data scatter around the parabolic trend in an order of magnitude of approx. 40 µm. The scattering occurs systematically reproducible within the specified random error of the triangulation sensor.

Figure 7c shows the fitted measurement data to the reference geometry of the tooth flank standard in the workpiece coordinate system measured by the confocal-chromatic sensor. Again, the blue dots illustrate the measurements, and the black line the reference geometry. The appropriate plumb line distances are plotted over the *x*-component of the measurement points (see Figure 7d). Here too, almost the entire tooth flank is measurable using the lateral scanning position approach. Based on the fitted measurement data to the reference geometry, a maximum tilt angle of −18.1° occurs at the tooth root. In gear measurements, thus, a larger angular range can be measured than specified. Note that the measurement value at *x* ≈ 94 mm was declared an outlier due to a significant deviation from the measured curve and removed for evaluation. The shape of the tooth flank standard measured with the confocal-chromatic sensor also deviates from the reference geometry. At the root as well as the tip of the tooth, the measured involute is more curved than the reference involute, similar to the behavior of the laser triangulation data. The results of the confocal-chromatic measurement scatter with a magnitude of around 10 µm within the specified random error of the sensor.

**Figure 8:**
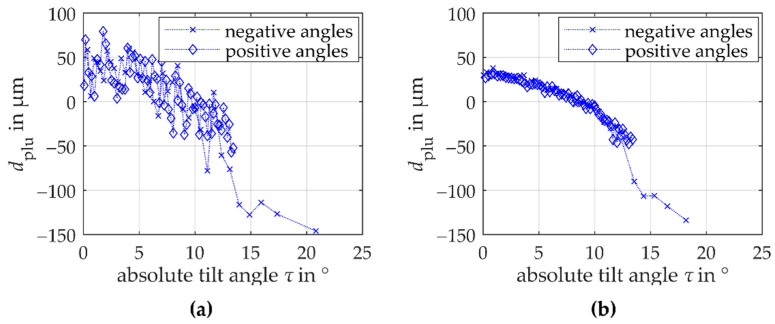
Dependence of the determined plumb line distances of (**a**) the triangulation gear shape measurements on the tooth flank standard and (**b**) the confocal-chromatic gear shape measurements on the tooth flank standard on the absolute values of the estimated tilt angles between the gear surface normal and the sensor axis. The crosses represent the tilt angles in the direction of the root (mathematically negative) and the diamonds represent the tilt angles in the direction of the tip (mathematically positive). From the symmetrical behavior of the plumb line distances it can be seen that no separate influence of the curvature can be observed within the scattering and that a significant dependence on the surface tilt can be expected.

Both the triangulation and the confocal-chromatic measurements systematically deviate parabolically from the involute reference geometry. Because a stronger curvature is perceived above all in the area of the root and the area of the tip (symmetrical behavior), the tilt angle between the surface normal and the sensors axis is assumed to have a significant systematic influence on the observed deviations. However, with the used measurement approach the influence of the surface tilt on the measurement deviations cannot be separated from a potential influence of the surface curvature (see Section 3.4). To qualitatively assess the effects of tilt and curvature, the symmetry behavior of the tilt angle around the center of the measured flank is exploited. Therefore, Figure 8 shows the dependence of the plumb line distances on the estimated absolute values of the tilt angles *τ* between the surface normal and the sensor axis. The tilt angles of triangulation and confocal-chromatic measurement are determined based on the fitted measurement data. Here, an almost perpendicular sensor alignment to the scanning unit’s axis is assumed. Thus, the calculated tilt angles are a mathematical estimate that appears geometrically plausible.

Figure 8a shows the plumb line distances for the laser triangulation measurement. The crosses represent the plumb line distances to the mathematically defined negative tilt angles in the direction of the tooth root and the diamonds represent the plumb line distances to the mathematically positive defines tilt angles in the direction of the tooth tip. If the absolute value of the tilt angle between the surface normal and the sensor axis increases in magnitude, the measuring deviation decreases to the negative µm range. A dependence on the angle sign is not seen within the scattering of the plumb line distances. Besides, within the scattering of the measurement data, no separate influence of the monotonically decreasing surface curvature from the root to the tip of the flank on the plumb line distances is detectable. Analogous to Figure 8a, Figure 8b illustrates the plumb line distances of the confocal-chromatic gear shape measurement depending on the absolute values of the tilt angles. Here too, the measurement deviation decreases to the negative µm-range when the absolute value of the tilt angle increases. Again, within the scattering of the plumb line distances no evidence for a separate influence of the surface curvature is observed.

Based on these results, the surface tilt seems to be the cause of the parabolic deviations, which corresponds to the theoretical contemplations. It is noticeable, however, that the deviation trend of the determined plumb line distances is comparable in shape and magnitude for both measurement methods. An influence by the surface tilt alone is therefore unlikely because both measurement methods are based on markedly different measuring principles. It is accordingly assumed that the parabolic deviation is influenced by another, unknown source of deviation. It is independent of the used measuring principles and can result, for example, from the fitting algorithm or the sensor alignment.

In summary, within the scatter of the plumb line distances of both the triangulation and the confocal-chromatic measurement, a separate effect of the varying surface curvature of the involute cannot be validated, which agrees to the theoretical contemplations. A parabolic systematic influence of the tilt angle between the surface normal and the sensors axis as well as an additional unknown source on the measurement deviation for both sensor principles is quantified and can therefore be considered for further uncertainty estimations.

#### 4.1.2. Random Deviations

For the study of the achievable measurement uncertainty due to random deviation sources in the gear shape measurements, the parabolic systematic deviation is mathematically eliminated. Therefore, a calibration based on both the triangulation and the confocal-chromatic measurement results is performed. The remaining scatter is assumed to primarily result systematically from the interaction with the surface properties such as the randomly changing local topography. To study the assumed influence of the surface topography, the results of comparison measurements at different heights on the tooth flank standard are discussed.

**Figure 9:**
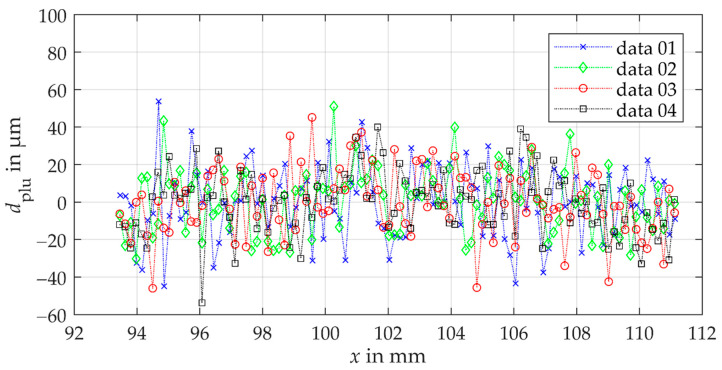
Remaining deviations of the corrected comparison triangulation gear shape measurements as a function of the x-component of the measurement point. The measurements are performed at different heights on the tooth flank standard to study the influence of the local topography of the surface on the measurement deviation.

Figure 9 shows the remaining plumb line distances of the corrected comparison measurements of the tooth flank standard using the laser triangulation sensor. The results of the comparison measurements show the sufficiency of the model-based compensation for the parabolic systematic deviation. Moreover, the comparison measurements of the tooth shape show a different scattering as a function of the measured x-position. This observed scattering between measurements at different heights shows a random deviation behavior, which agrees with the assumption about the surface topography influence. Thus, the standard measurement uncertainty from random sources of the optical laser triangulation gear shape measurement can be calculated from the empirical standard deviation of the determined random deviations (see Figure 3). The achieved standard measurement uncertainty of the triangulation measurements amounts to 18 µm. Based on this result, the measurement approach using the laser triangulation sensor currently does not achieve the required measurement uncertainty of <10 µm even by only considering the random deviation sources. Besides, a maximum deviation of ≈55 µm occurs. The results show that the specified measurement deviation (linearity error) of the triangulation sensor of ±30 µm cannot be maintained for gear shape measurements.

**Figure 10:**
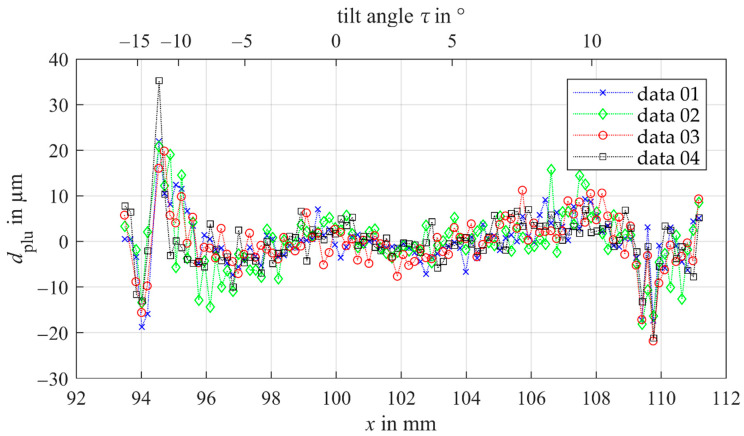
Remaining deviations of the corrected comparison measurements of the gear shape using the confocal-chromatic sensor as a function of the x-component of the measurement point. A second *x*-axis illustrates the dependency of the plumb line distances of the tilt angles. The measurements are performed at different heights on the tooth flank standard to study the influence of the local topography of the surface on the measurement deviation.

In Figure 10 the remaining plumb line distances of confocal-chromatic gear shape measurements at different heights on the tooth flank standard are plotted as a function of the x-component of the measuring points. A second nonlinear *x*-axis illustrates the dependency of the plumb line distances of the tilt angles. Here too, the systematic parabolic trend is compensated successfully. The pattern of the scatter as a function of the measured x-position mainly differs randomly for the comparison measurements of the tooth shape. Thus, a random error due to random surface properties (topography) also primarily influences the achievable measurement uncertainty of the gear shape measurement using a confocal-chromatic sensor. With the confocal-chromatic sensor, a standard measurement uncertainty of 6.2 µm is achievable. If the surface tilt exceeds angles of ±10°, mainly systematic measurement deviations of ±20 µm occur, which probably result from the proximity of the tilt angle to the limit of the sensor’s acceptance angle of ±17°. The specified linearity error of 2.5 µm of the confocal-chromatic sensor, therefore, does not apply to gear shape measurements.

In summary, the observed random deviations for the comparison measurements at different heights of the tooth flank standard verify the hypothesis that surface properties do have a significant influence on the random deviations. Besides, systematic deviations at the edge of the acceptance angle range influence the achievable uncertainty. The results show the potential of confocal-chromatic sensors for gear shape measurements because the achieved standard measurement uncertainty for random deviation contributions meets the required measurement uncertainty of <10 µm.

### 4.2. Spur Gear Measurements

**Figure 11:**
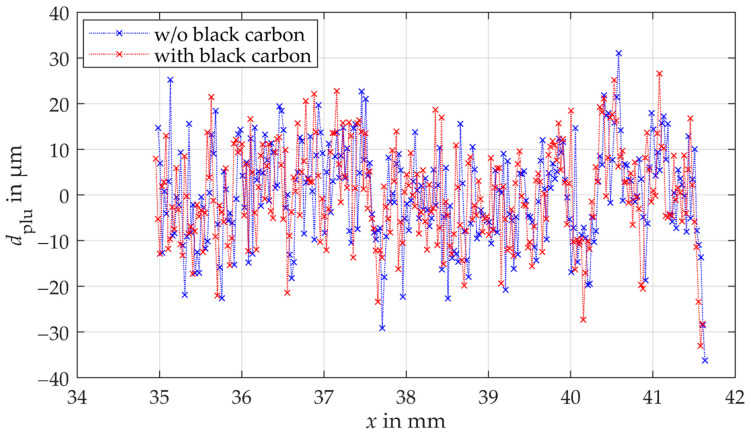
Plumb line distances of the comparison measurements of the gear shape using the triangulation sensor. The blue crosses present the measurement without the coated adjacent tooth. The red crosses are the results of the measurement with the coated adjacent.

The effect of multiple reflections on the measurement deviation and thus the achievable measurement uncertainty is investigated with comparison measurements on a spur gear with and without covering the adjacent tooth flank with a black carbon layer. The tooth flank is scanned laterally with a lateral resolution of 0.025 mm, resulting in a total of up to 267 measuring points. Per measuring point 100 distance measurements are performed. The results of the plumb line distances of a comparison measurement using the triangulation sensor are shown in Figure 11. Although the triangulation sensor is only specified for a maximum acceptance angle of 30°, it was possible to measure almost the entire tooth flank (maximum acceptance angle of >30° required). The results of the comparison measurements using the laser triangulation sensor indicate no significant influence of multiple reflections on the measurement uncertainty (see Figure 11). Both measurements scatter with the same order of magnitude. The standard measurement uncertainty of the tooth shape measurements with a black carbon coated adjacent tooth is −0.1 µm smaller than the standard measurement uncertainty without the coated adjacent tooth. The deviation of −0.1 µm is within the specified reproducibility of the sensor (see Table 2) and is therefore negligible. Only the calculated plumb line distances vary locally, although almost the same points of the gear surface are measured during the comparison measurements. A cause for the local deviations could be the invasive application of the carbon black coating to the adjacent tooth. The measured points on the tooth flank could therefore differ slightly in the comparison measurements.

**Figure 12:**
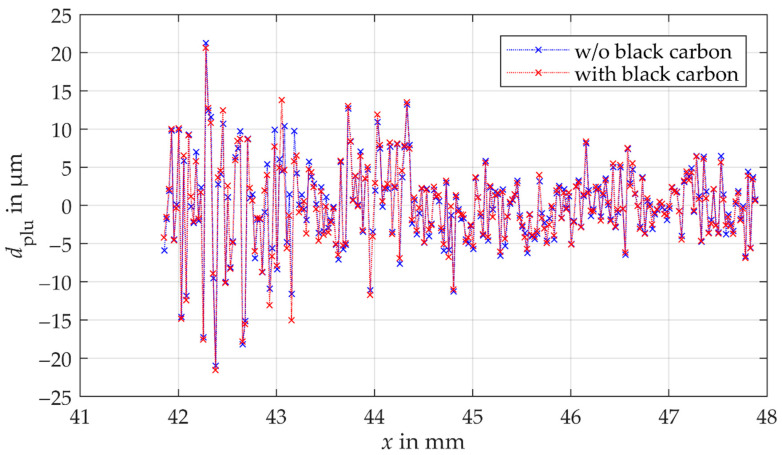
Plumb line distances of the comparison measurements of the gear shape using the triangulation sensor. The blue crosses present the measurement without the coated adjacent tooth. The red crosses are the results of the measurement with the coated adjacent.

The determined plumb line distances of the comparison measurements using the confocal-chromatic distance sensor are illustrated in Figure 12. Here, too, no influence of multiple reflections can be observed on the standard measurement uncertainty. The results of the measurements scatter within the same order of magnitude. Moreover, the shape of the plumb line distances is comparable. The standard measurement uncertainty of the measurements with the coated adjacent tooth is 0.04 µm larger than the standard measurement uncertainty of the measurements without the coated adjacent tooth. The relative deviation of 0.04 µm is within the reproducibility of the confocal-chromatic sensor.

In summary, the results for the laser triangulation sensor and the confocal-chromatic sensor show that multiple reflections do not influence significantly the tooth shape measurement with a lateral scanning position measurement approach.

## 5. Conclusions and Outlook

In this article, the potential of a commercial triangulation sensor and a commercial confocal-chromatic sensor for the measurement of gears is studied. In addition, the sources of deviations such as the tilt angle between the gear surface normal and the sensor axis, the varying surface curvature, and the gear surface topography are examined. For that purpose, measurements on a tooth flank standard and a small spur gear are performed with both measurement principles. The comparison measurements on a small spur gear show that the standard measurement uncertainty in optical gear shape measurements is not affected by multiple reflections. However, a systematic parabolic deviation to the reference geometry is observed in both the triangulation and the confocal-chromatic gear shape measurements on the tooth flank standard. Due to the symmetry of the parabolic deviation, an influence of the monotonically varying surface curvature of the involute can be neglected. The deviation can partly be attributed to an influence of the surface tilt, but not fully explained. Hence, an unknown source of deviation is assumed, which is expected to result from the fitting procedure to calculate the plumb line deviations of the measurements from the reference geometry. To eliminate the parabolic systematic deviation, a calibration based on both the triangulation and the confocal-chromatic measurements is performed. A randomly scattering deviation due to the local topography dominates the achieved residual deviations for both sensors.

To assess the potential of the laser triangulation sensor, the achievable standard measurement uncertainty from random sources is calculated to 18 µm from the residual deviations, which does not comply with the requirements of a single-digit µm uncertainty. Even if the entire tooth flank of the tooth flank standard is measurable with the laser triangulation sensor, maximum deviations of 55 µm occur and the specification of the linearity error of the triangulation sensor do not apply to gear shape measurements.

The achievable standard measurement uncertainty due to random sources of the confocal-chromatic gear shape measurement of the tooth flank standard amounts to <6.5 µm. In addition, a systematic deviation at the edges of the acceptance angle range superimposes with the random deviation. If the tilt angles exceed ±10°, local deviations of ±20 µm occur. Although the determined uncertainty is higher than the value of the linearity error of the confocal-chromatic sensor, the obtained results meet the requirements for gear shape measurements and show potential for optical gear shape measurements using confocal-chromatic sensors.

In summary, the potential of optical sensors for gear shape measurements is demonstrated. Particularly with the commercial confocal-chromatic sensor a measurement uncertainty <10 µm is feasible, although the sensor is not (yet) designed for optical gear shape measurements.

To further reduce the random measurement uncertainty contribution, the interaction of the sensor principles with the surface must be understood. Measurements on defined tooth surfaces with different topography and roughness have to be performed in the future. The parabolic systematic deviation must also be investigated in future steps to clarify whether the surface tilt is responsible for it or to identify the real source. Currently, it is assumed that the parabolic shape of the systematic deviation results mainly from the approximation process and only to a minor extent from the surface tilt angle. The approximation algorithm has to be adapted in this respect. Another systematic deviation occurs if the perpendicular alignment between the sensor and the linear unit as well as the alignment of the sensor axis within the gear transverse section is not successful. Suitable adjustment and calibration processes must be investigated to determine their contributions to the total uncertainty budget.

## Figures and Tables

**Table 1:** Geometric parameters of the tooth flank standard and small spur gear.

	Number of Teeth	Normal Module in mm	Base Circle Diameter in mm
tooth flank standard	20	10.64117	199.99
spur gear	26	3.75	91.62

**Table 2:** Specifications of the confocal-chromatic sensor and the laser triangulation sensor. The reproducibility of the confocal-chromatic distance sensor is determined experimentally because no value of the reproducibility is specified in the datasheet.

	Laser Triangulation Sensor	Confocal-Chromatic Sensor
measuring range	50 mm	10 mm
acceptance angle	±30°	±17°
light spot diameter *w*_d_	55–570 µm	16 µm
linearity error	±30 µm	±2.5 µm
reproducibility	±2 µm	±0.3 µm

**Table 3:** Maximum distance variation ∆*d* as a function of the light spot diameter *w*_d_ of the sensors and the maximum estimated tilt angle of approx. −18° between the surface normal and the sensor axis and the varying surface curvature, respectively. The results are based on the geometry of the tooth flank standard and the lateral scanning position measurement approach used.

	Laser Triangulation Sensor (Mid-Range) *w*_d_ = 55 µm	Confocal-Chromatic Sensor *w*_d_ = 16 µm
∆*d*_tilt_ in µm	17.9	5.2
∆*d*_curvature_ in µm	0.04	0.003

## Data Availability

Data will be made available on request.
